# Supplementary material for: Predicting individual patient and hospital-level discharge using machine learning
Source: Commun Med (Lond). 2024 Nov 18;4:236. doi: 10.1038/s43856-024-00673-x (PMC11574281; doi:10.1038/s43856-024-00673-x)
Supplement: Supplementary file 2 — Supplementary Information [file 43856_2024_673_MOESM2_ESM.pdf]

Predicting individual patient and hospital-level discharge using machine learning: supplementary material

Jia Wei, Jiandong Zhou, Zizheng Zhang, Kevin Yuan, Qingze Gu, Augustine Luk, Andrew J Brent, David A. Clifton, A. Sarah Walker, David W. Eyre

Contents

- Supplementary Methods
- Supplementary Figures 1-13
- Supplementary Tables 1-3

Supplementary Methods

A total of 1,152 features were created and grouped into 16 feature categories:

1. Index date (4 features):

- Number of days since 1 January 2017
- Index date weekday
- Index date month
- Number of hours since admission

2. Demographics (13 features):

- Age
- Sex
- BMI, height, weight
- Ethnic group (white, mixed, Asian or Asian British, Black or Black British, other ethnic group, not stated/not known ethnic group)
- IMD score
- Prior mean, max, min, median, sd length of stay by postcode district

3. Comorbidities (52 features):

- Charlson comorbidity index, raw and age-adjusted
- Elixhauser comorbidity score, raw and age-adjusted
- Individual comorbidities in Charlson score (acute myocardial infarction, cerebral vascular disease, congestive heart failure, connective tissue disorder, dementia, diabetes, liver disease, peptic ulcer, peripheral vascular disease, pulmonary disease, cancer, diabetes complications, paraplegia, renal disease, metastatic cancer, severe liver disease, HIV)
- Individual comorbidities in Elixhauser score (congestive heart failure, cardiac arrhythmias, valvular disease, pulmonary circulation disorders, peripheral vascular disorders, hypertension uncomplicated, paralysis, other neurological disorders chronic pulmonary disease, diabetes uncomplicated, diabetes complicated, hypothyroidism, renal failure, liver disease, peptic ulcer disease, HIV, lymphoma, metastatic cancer, solid tumour without metastasis, rheumatoid arthritis, coagulopathy, obesity, weight loss, fluid and electrolyte disorders, blood loss anaemia, deficiency anaemia, alcohol abuse, drug abuse, psychoses, depression, hypertension complicated)

All comorbidities are based on diagnostic codes in previous year before the current admission.

4. Current admission (7 features):

- Admission time/source features:
  - daytime (0 to 24 hours)
  - admission weekday (Monday to Sunday)
  - admission month (January to December)
  - admission source (usual place of residence, non-NHS institutional care, other NHS Provider)
- Admission specialty:

Acute, emergency and geriatric medicine; Acute and general surgery; Trauma and orthopaedics; Critical care; Medical subspecialty; Surgical subspecialty; Others.

  - Specialty at index date
  - Number of each specialty in current admission
  - Number of unique specialties admitted within 365 days before the index date
- Number of new specialties under within last 24/48 hours

5. Ward stay (4 features):

- Number of new wards within 24/48 hours
- The current ward is ICU
- Hours elapsed since the current ward starts

6. Diagnosis (8 features):

Length of stay statistics for previous admissions for all patients, by SHMI category

- LOS characteristics of SHMI diagnosis categories: Historic mean/median/maximum/minimum/SD of the LOS of patients within the same SHMI diagnostic category

7. Discharge planning (3 features):

- Physiotherapy referral within 24/48 hours before index date/ within 365 days before current admission date

8. Procedures (21 features):

- Had procedure within 24/48 hours before index date/within current admission
- Number of procedures 24/48 hours before index date/within current admission*
- Time elapsed since most recent procedure before the index date, days
- Had procedure within 365 days before current admission date
- Number of procedures within 365 days before the current admission date*

* Procedures were identified using OPCS (Operating Procedure Codes Supplement) codes. We excluded modifying codes starting with 'Y' and 'Z' to make sure each procedure was counted just once.

9. Antibiotics prescriptions (73 features):

- Current antibiotic use within 24/48 hours before the index date
- New antibiotics within 24/48 hours before the index date
- Antibiotics completed within 24/48 hours before index date
- Any antibiotics used within the current admission
- Duration of antibiotics used within current admission
- Count of unique antibiotics in the current admission
- Currently on a specific antibiotics agent (~60 antibiotics)

10. Medication (36 features):

- Use of intravenous fluids/intravenous medication/oral medication/nebulised medication/inhalation medication within 24/48 hours before index date/within current admission

- Volume of intravenous fluids within 24/48 hours before index date/within current admission
- Count of intravenous medication/oral medication/nebulised medication/inhalation medication within 24/48 hours before index date/within current admission
- Use of intravenous/oral strong opiates within 24/48 hours before index date/within current admission

11. Microbiology tests (6 features):

- Extended Spectrum Beta-Lactamase (ESBL)/ Carbapenemase-producing. Enterobacteriaceae (CPE) isolated in the current admission
- Vancomycin-resistant enterococci (VRE) isolated in the current admission
- Methicillin-resistant Staphylococcus aureus (MRSA) isolated in the current admission
- ESBL/CPE isolated in the last 365 days
- VRE isolated in last 365 days
- MRSA isolated in last 365 days
- Positive blood culture results within 24/48 hours before index date/within current admission

12. Radiology investigation (9 features):

- Had radiology-based procedure within 24/48 hours before index date/within current admission
- Number of radiology-based procedures within 24/48 hours before index date/within current admission
- Had radiology-based procedure within 365 days before current admission date
- Number of radiology-based procedures within 365 days before the current admission date
- Time elapsed since most recent radiology procedure within 365 days before the index date

13. Readmissions and previous hospital stay (22 features):

- Readmissions
- − Current admission is early readmission: ≤30 days from a previous hospitalization event
- − Current admission is late readmission: >30 to ≤180 days from a previous hospitalization event
- − Number of early 30-day readmissions within 365 days before the index date
- − Time elapsed from most recent early 30-day readmission within 365 days before the index date, days
- Previous length of stay
    - Number of admissions within 30/90/365 days before the index date
    - Cumulative LOS within 30/90/365 days before the index date
    - Mean/Maximum/Minimum/SD LOS per admission within 30/90/365 days before the current admission date

14. Hospital capacity factors (23 features):

- The median/mean/maximum/minimum/SD LOS of the current ward
- The current number of inpatients in the hospital

- Current inpatients with LOS to date of >= 7 days, 14 days, 28 days
- Proportion of current inpatients with LOS to date of >= 7 days, 14 days, 28 days
- Number of admissions within the last 24hr, 48hr, 7d, 28d
- Number of discharges within the last 24h, 48h, 7d, 28d
- The mean LOS for all discharges in the 7d, 14d, 28d before the index date

15. Vital signs (135 features):

- Mean / Max / Min / SD of each vital sign measurements (see below) within 24/48 hours before index date/within current admission
- Number of vital signs measurements within 24/48 hours before index date/within current admission

List of vital signs: heart rate, respiratory rate, systolic blood pressure, diastolic blood pressure, temperature, oxygen saturation, O2 L/min, O2 delivery device, AVPU score, NEWS2 score, NEWS2 score alternative (missing oxygen device = Room air)

16. Laboratory tests (736 features):

- Mean / Max / Min / SD of each laboratory test measurements within 24/48 hours before index date/within current admission
- Number of laboratory test measurements within 24/48 hours before index date/within current admission/within 365 days before the index date

List of laboratory tests:

- **Complete blood counts:** Haemoglobin, Haematocrit, Mean Cell Volume, White Cell Count, Platelets, Neutrophils, Lymphocytes, Eosinophils, Monocytes, Basophils
- **Renal functions:** Creatinine, Urea, Potassium, Sodium, EGFR, Bicarbonate
- **Inflammatory:** C-reactive protein, Erythrocyte Sedimentation Rate, Creatinine kinase
- **Liver functions:** Alkaline phosphatase, Aspartate aminotransferase, Alanine transaminase, Albumin, Bilirubin, Amylase, Gamma-glutamyl Transferase
- **Bone/electrolytes profiles:** Adjusted calcium, Magnesium, Phosphate
- **Clotting:** Activated partial thromboplastin time, Prothrombin time, D_dimer,
- **Endocrine:** Thyroid-stimulating hormone, HbA1c, Glucose, Prostate-specific antigen
- **Haematinics:** Ferritin, Iron, Transferrin, B12, Folate
- **Others:** Lactate dehydrogenase, Troponin, Total Ig
- **Blood gases:** Base excess, Partial pressure of oxygen, Partial pressure of carbon dioxide, Lactate, Arterial blood pH
- **Lipids:** Triglycerides, High-density lipoprotein cholesterol, Total cholesterol, Low-density lipoprotein cholesterol

Supplementary Figures

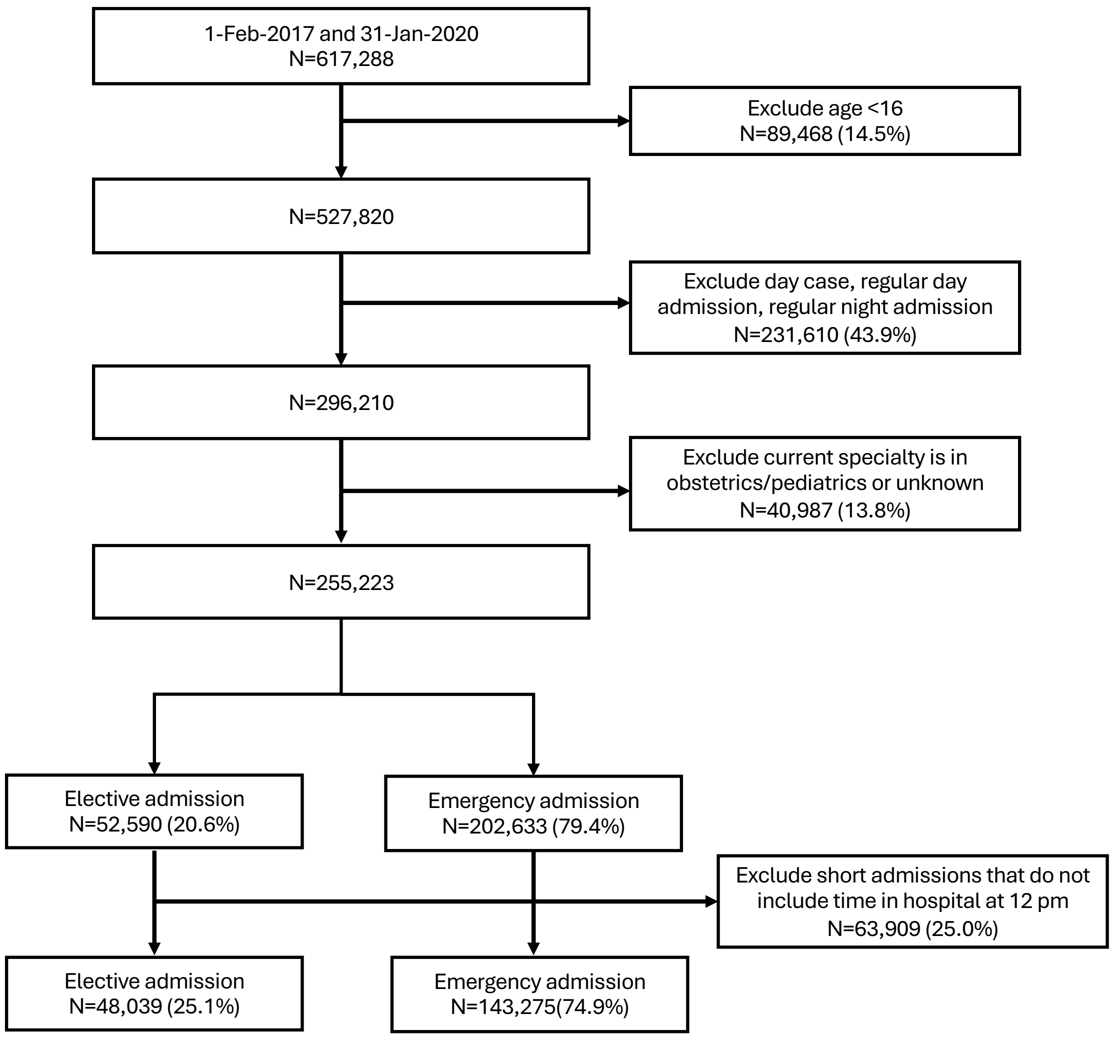

Supplementary Figure 1. Study inclusion and exclusion flow chart.

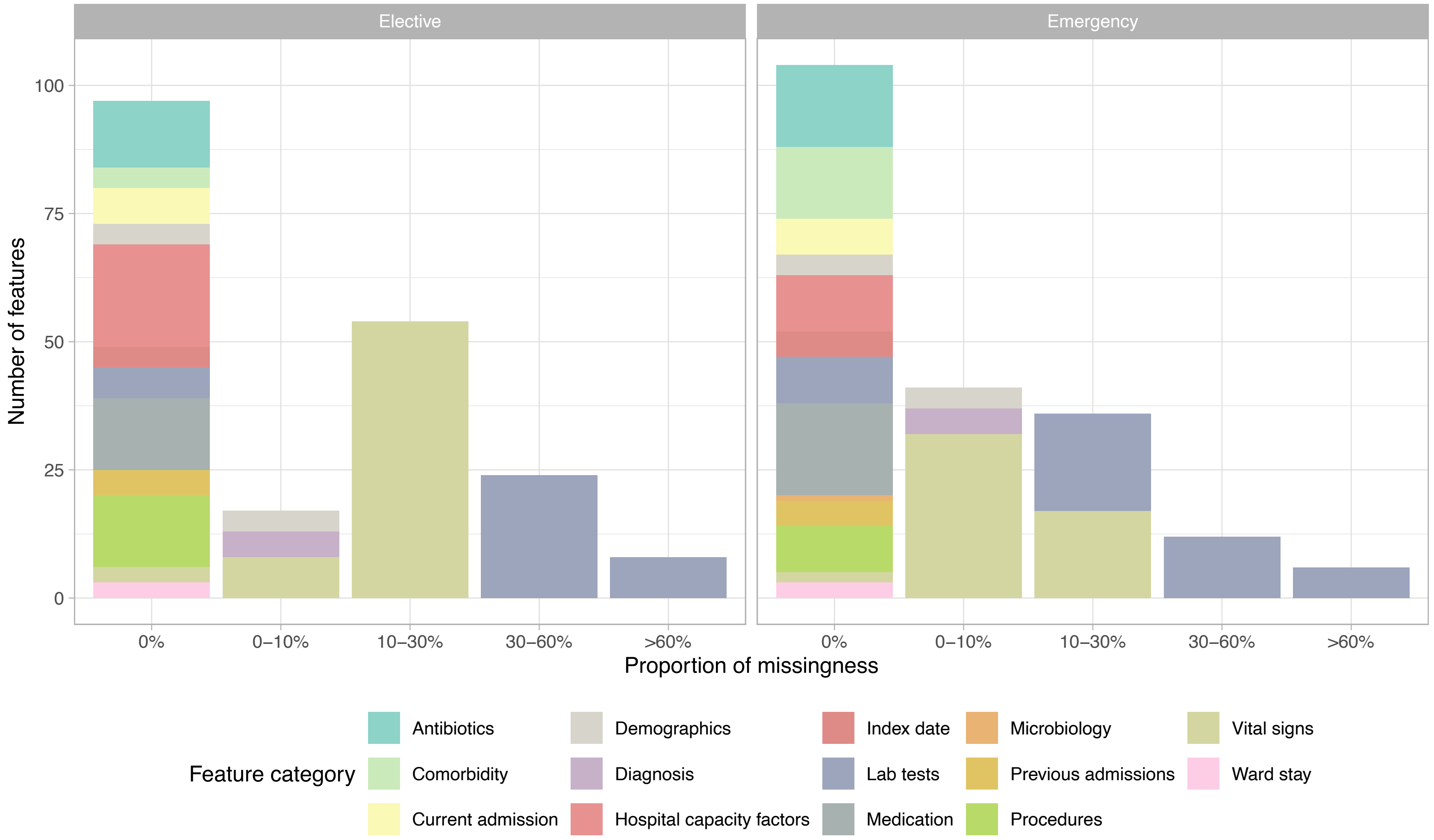

Supplementary Figure 2. Number of features with missing values by proportion of missingness and feature category. SHMI indicates diagnosis (length of stay statistics for previous admissions for all patients, by SHMI category).

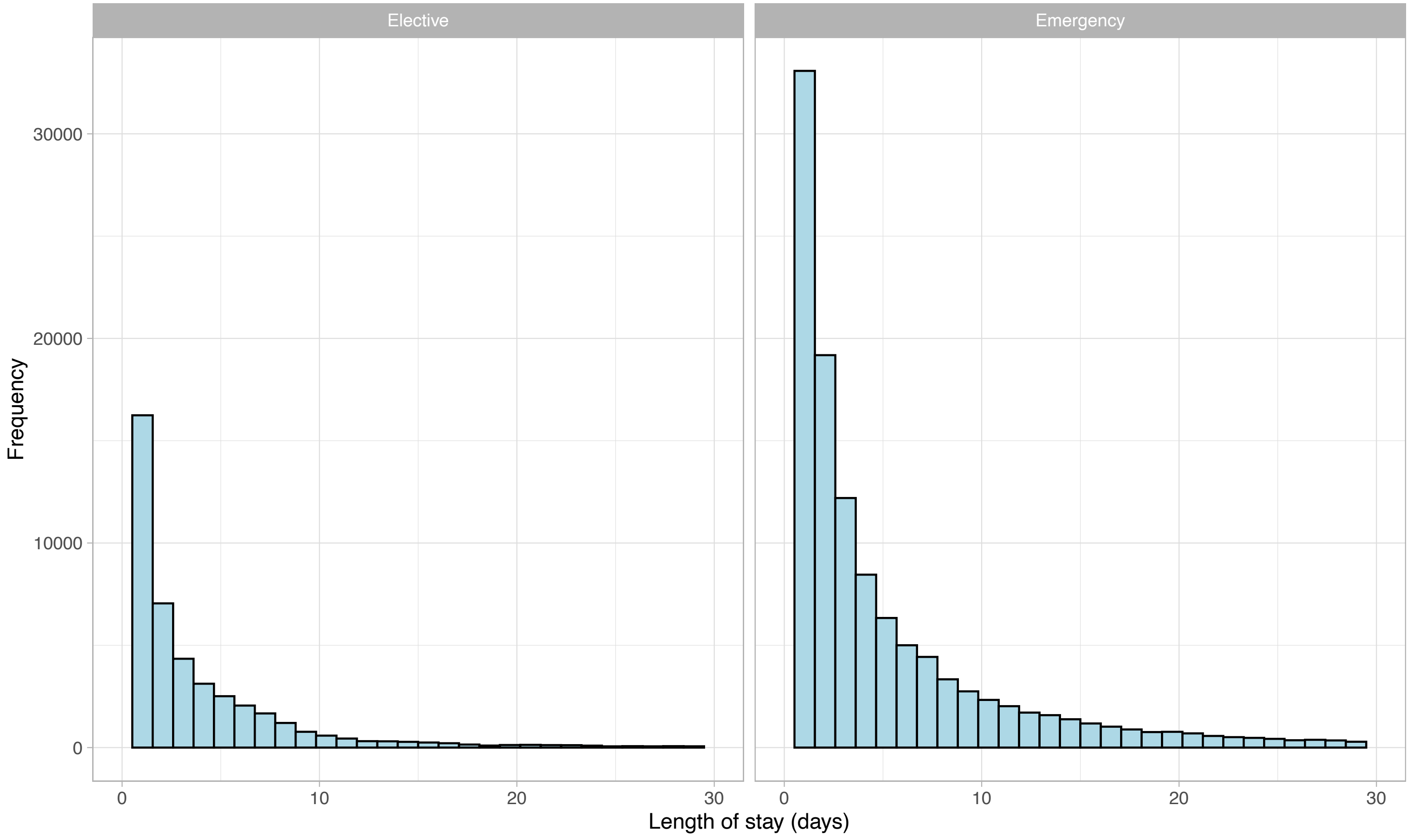

Supplementary Figure 3. Distribution of length of stay (from admission to discharge) for elective and emergency patients. Length of stay is censored at 30 days for better visualisation (712 elective admissions and 4212 emergency admissions had a length of stay >30 days).

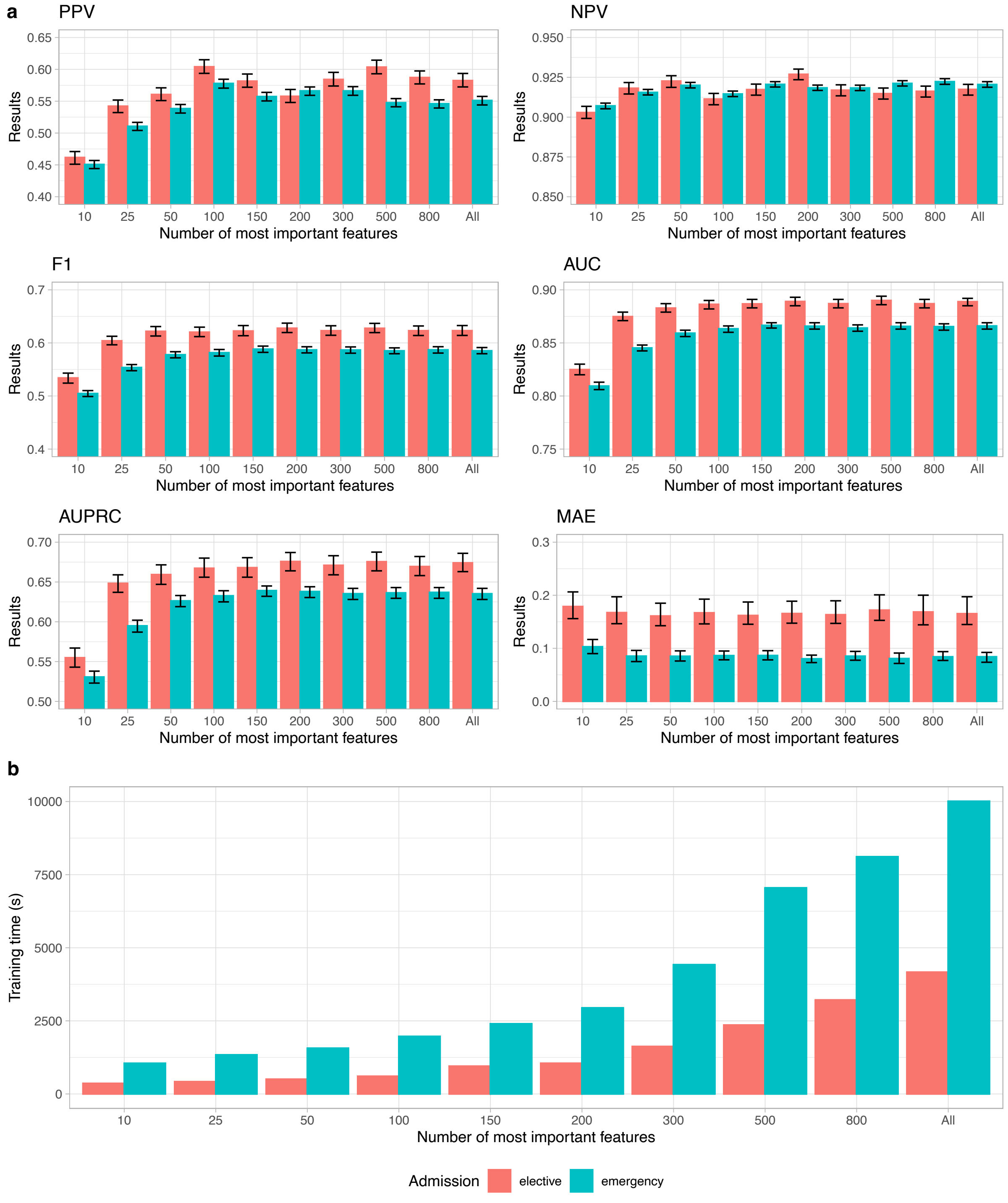

Supplementary Figure 4. Impact of number of features on model performance (a) and training time (b). The top 10, 25, 50, 100, 150, 200, 300, and 500 most important features from the main models predicting elective and emergency discharges were selected and used for model training, respectively. 'All' included 1,152 features in total. AUC: area under the receiver operating curve; AUPRC: area under the precision-recall curve; MAE: normalised mean absolute error (mean difference in predicted and actual discharges per day divided by the mean number of discharges per day).

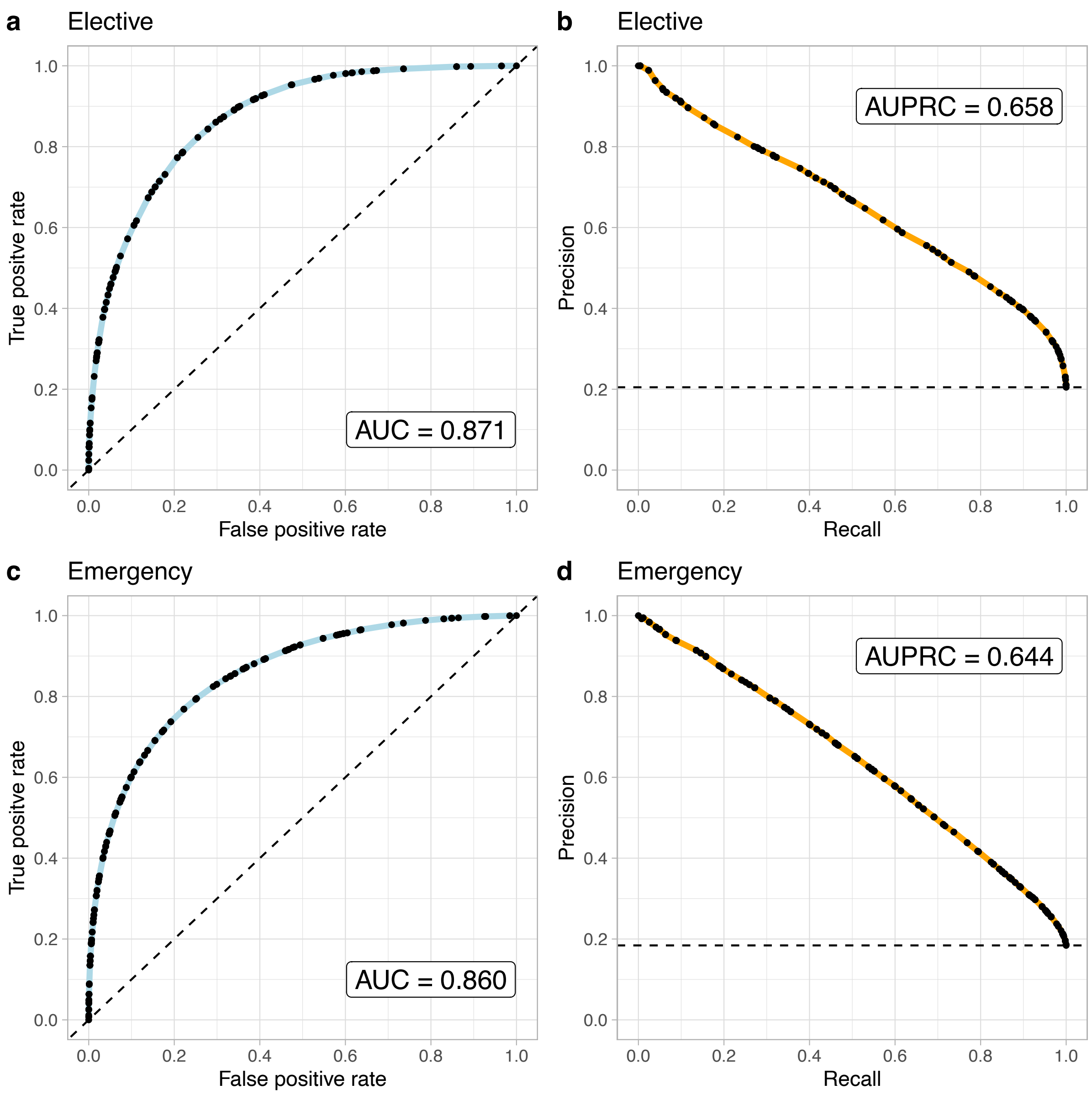

Supplementary Figure 5. Model performance of the extreme gradient boosting models in the test dataset (01 February 2019 to 31 January 2020). a) Area under the receiver operating curve (AUC) for elective admissions. b) Area under the precision-recall curve (AUPRC) for elective admissions. c) AUC for emergency admissions. d) AUPRC for emergency admissions.

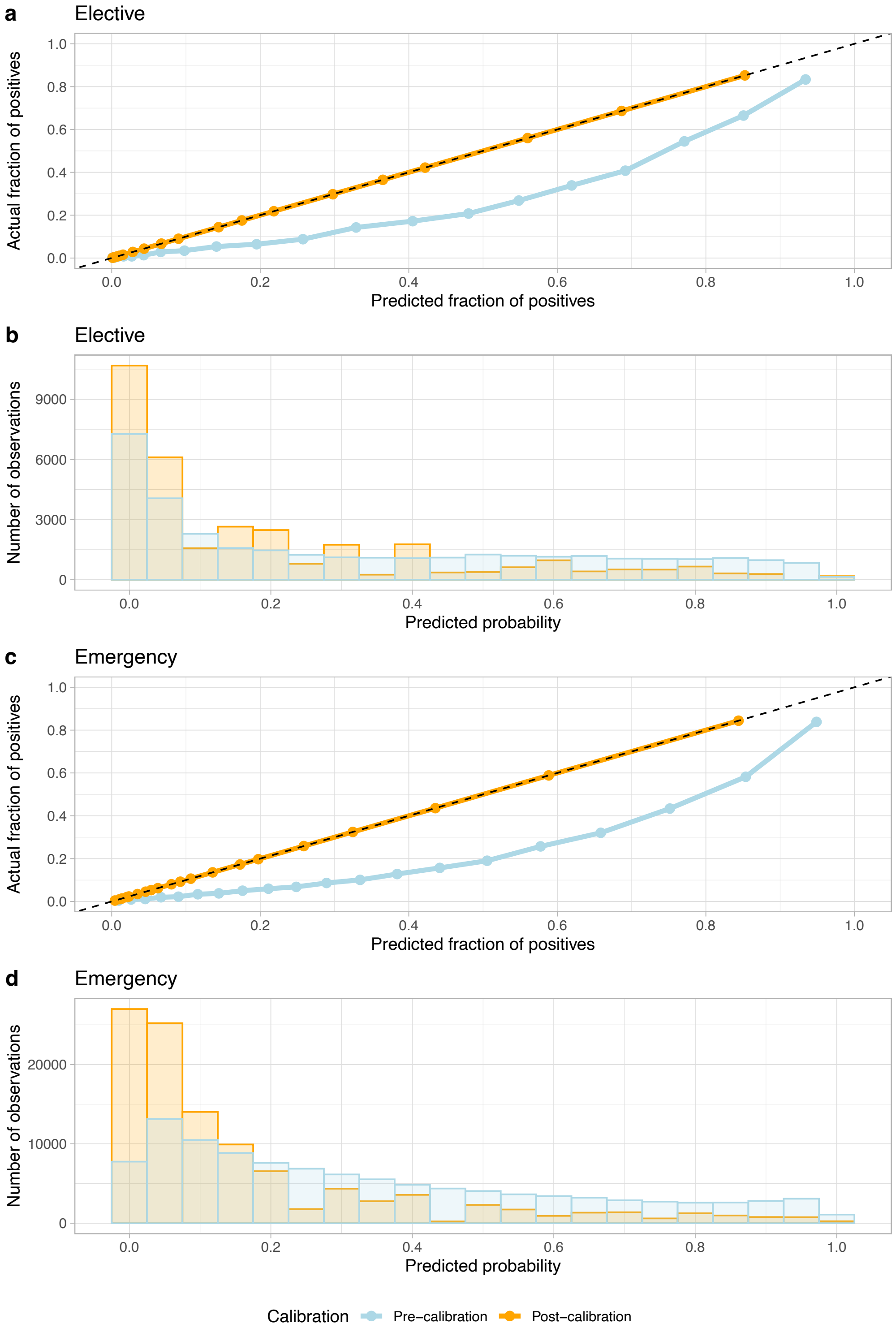

Supplementary Figure 6. Calibration curve for the extreme gradient boosting models using the validation dataset for elective admissions (a) and emergency admissions (c), and distribution of predicted probabilities pre- and post-calibration for elective admissions (b) and emergency admissions (d). The calibration error was 0.152 and 0.003 pre/post-calibration for elective admission, and 0.203 and 0.001 pre/post-calibration for emergency admission, respectively.

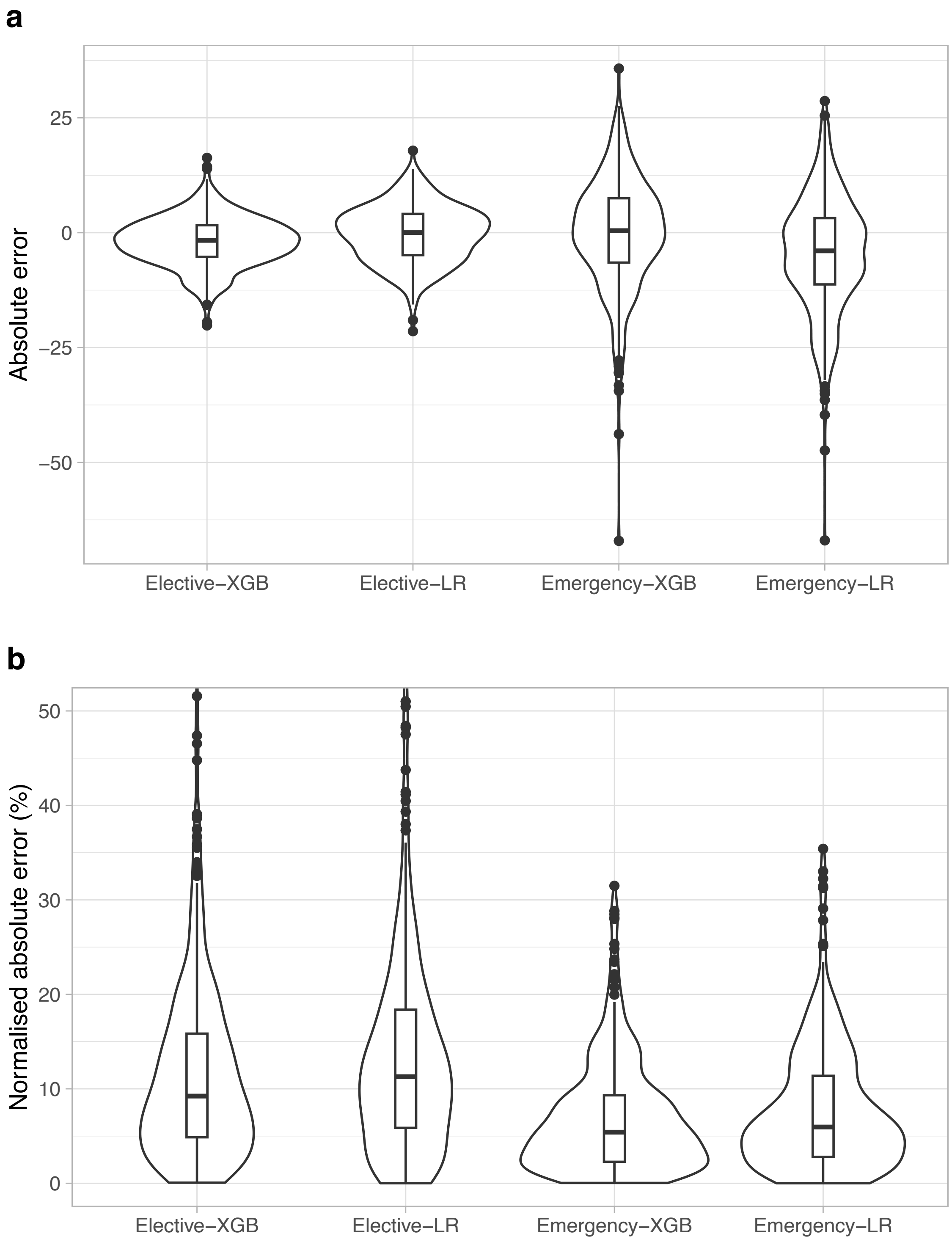

Supplementary Figure 7. Distribution of differences between the predicted total discharges and actual discharges for elective and emergency admissions using extreme gradient boosting (XGB) and logistic regression (LR) models. a, absolute error (differences in predicted and actual discharges each day); **b**, normalised absolute error (differences in predicted and actual discharges each day divided by the actual discharges). The box and whisker plots indicate the median and interquartile range.

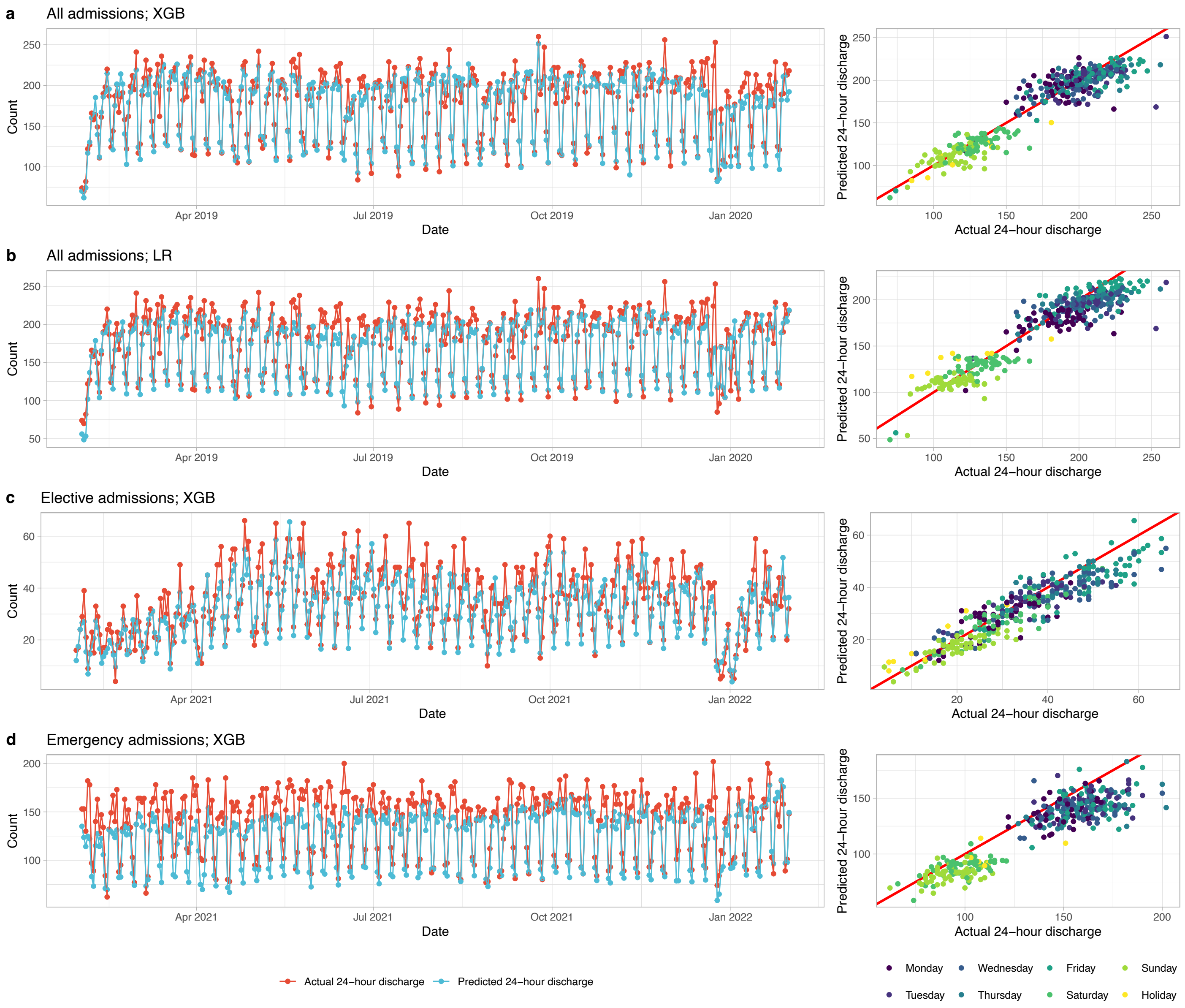
a
All admissions; XGB
b
All admissions; LR
c
Elective admissions; XGB
d
Emergency admissions; XGB
Count
Date
Apr 2019
Jul 2019
Oct 2019
Jan 2020
Apr 2021
Jul 2021
Oct 2021
Jan 2022
Predicted 24−hour discharge
Actual 24−hour discharge
Actual 24−hour discharge
Predicted 24−hour discharge
Monday
Tuesday
Wednesday
Thursday
Friday
Saturday
Sunday
Holiday

Supplementary Figure 8. Sensitivity analyses, predicted and actual number of discharges within 24 hours by calendar time in the test dataset. a) All admissions (elective and emergency) using a single extreme gradient boosting (XGB) model in the test dataset (01 February 2019 to 31 January 2020)**.** b) All admissions using baseline logistic regression (LR) model in the test dataset (01 February 2019 to 31 January 2020). c) Elective admissions using XGB model in the post-COVID test dataset (01 February 2021 to 31 January 2022). d) Emergency admissions using XGB model in the post-COVID test dataset (01 February 2021 to 31 January 2022).

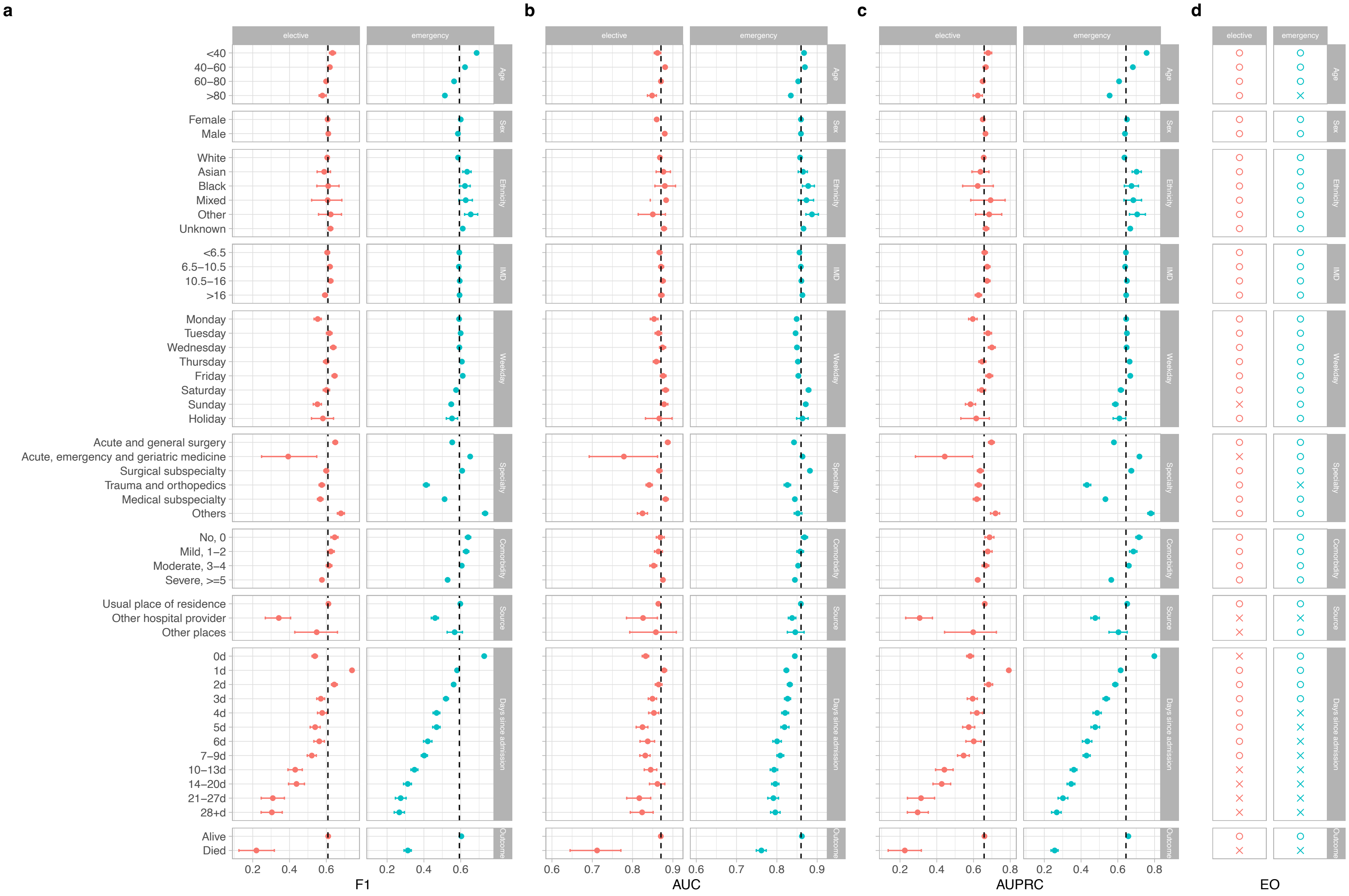
a
b
c
d
elective
emergency
Age
Sex
Ethnicity
IMD
Weekday
Specialty
Comorbidity
Source
Days since admission
Outcome
<40
40–60
60–80
>80
Female
Male
White
Asian
Black
Mixed
Other
Unknown
<6.5
6.5–10.5
10.5–16
>16
Monday
Tuesday
Wednesday
Thursday
Friday
Saturday
Sunday
Holiday
Acute and general surgery
Acute, emergency and geriatric medicine
Surgical subspecialty
Trauma and orthopedics
Medical subspecialty
Others
No, 0
Mild, 1–2
Moderate, 3–4
Severe, >=5
Usual place of residence
Other hospital provider
Other places
0d
1d
2d
3d
4d
5d
6d
7–9d
10–13d
14–20d
21–27d
28+d
Alive
Died
0.2 0.4 0.6
F1
0.6 0.7 0.8 0.9
AUC
0.2 0.4 0.6 0.8
AUPRC
EO

Supplementary Figure 9. Additional model performance indicators by subgroups in the test dataset (01 February 2019 to 31 January 2020). F1 score (a), area under the receiver operating curve (AUC) (b), and area under the precision-recall curve (AUPRC) (c) were compared. IMD=index of multiple deprivation score (higher scores indicate greater deprivation). Weekday refers to the day of the week of the index date. Source refers to the source of admission. Overall performance is shown by the dashed line in each plot. 95% confidence intervals were calculated using bootstrap. Balanced accuracy, positive predictive value (PPV), and negative predictive value (NPV) are shown in **Figure 3.** (d) Equalised odds (EO) differences for assessing model fairness, determined by either the per subgroup true positive rate or true negative rate differed from the overall rate by greater than an illustrative threshold of 0.1. 'O' represents a value ≤0.1 while 'X' represents a value >0.1.

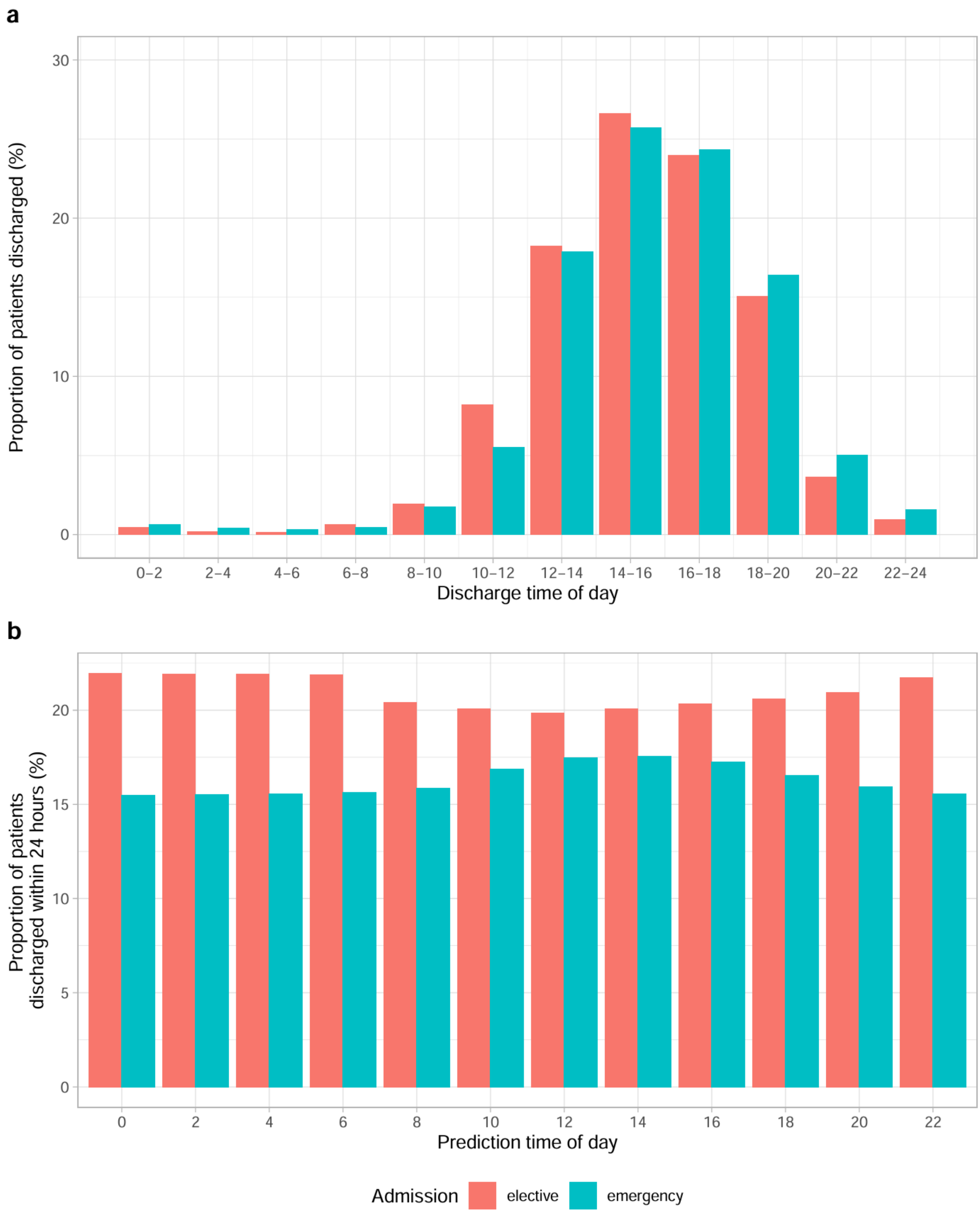

Supplementary Figure 10. Proportion of patients actually discharged by the observed hour of day of discharge (a) and proportion of patients discharged within the following 24 hours by model prediction time (b).

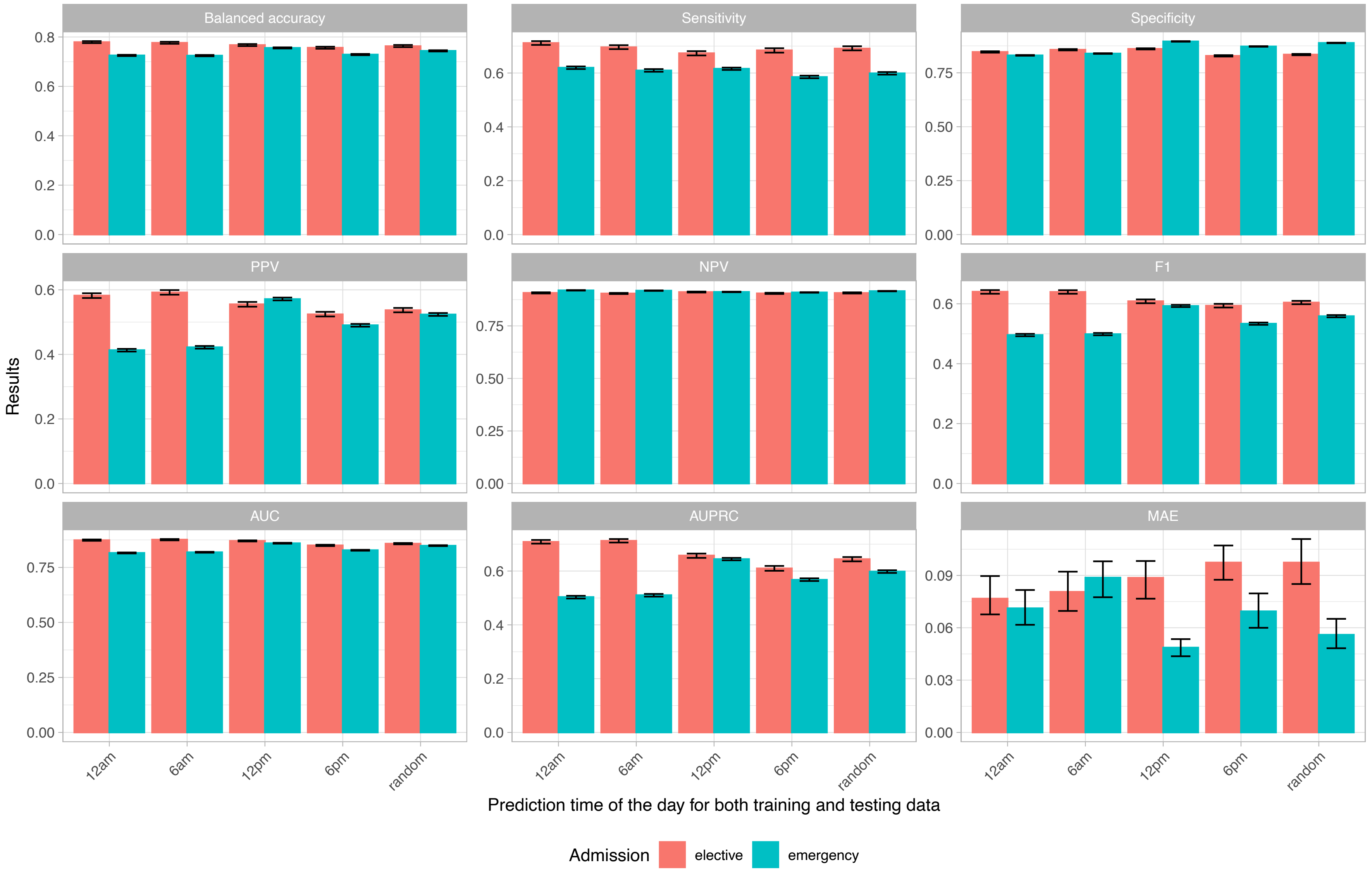

Balanced accuracy
Sensitivity
Specificity
PPV
NPV
F1
AUC
AUPRC
MAE
Results
12am
6am
12pm
6pm
random
Prediction time of the day for both training and testing data
Admission
elective
emergency

Supplementary Figure 11. Model performance using different prediction times of the day for elective and emergency admissions in the test dataset (01 February 2019 to 31 January 2020). Predictions are shown for models trained at the same time of day. PPV: positive predictive value; NPV: negative predictive value; AUC: area under the receiver operating curve; AUPRC: area under the precision-recall curve; MAE: normalised mean absolute error (mean difference in predicted and actual discharges per day divided by the mean number of discharges per day).

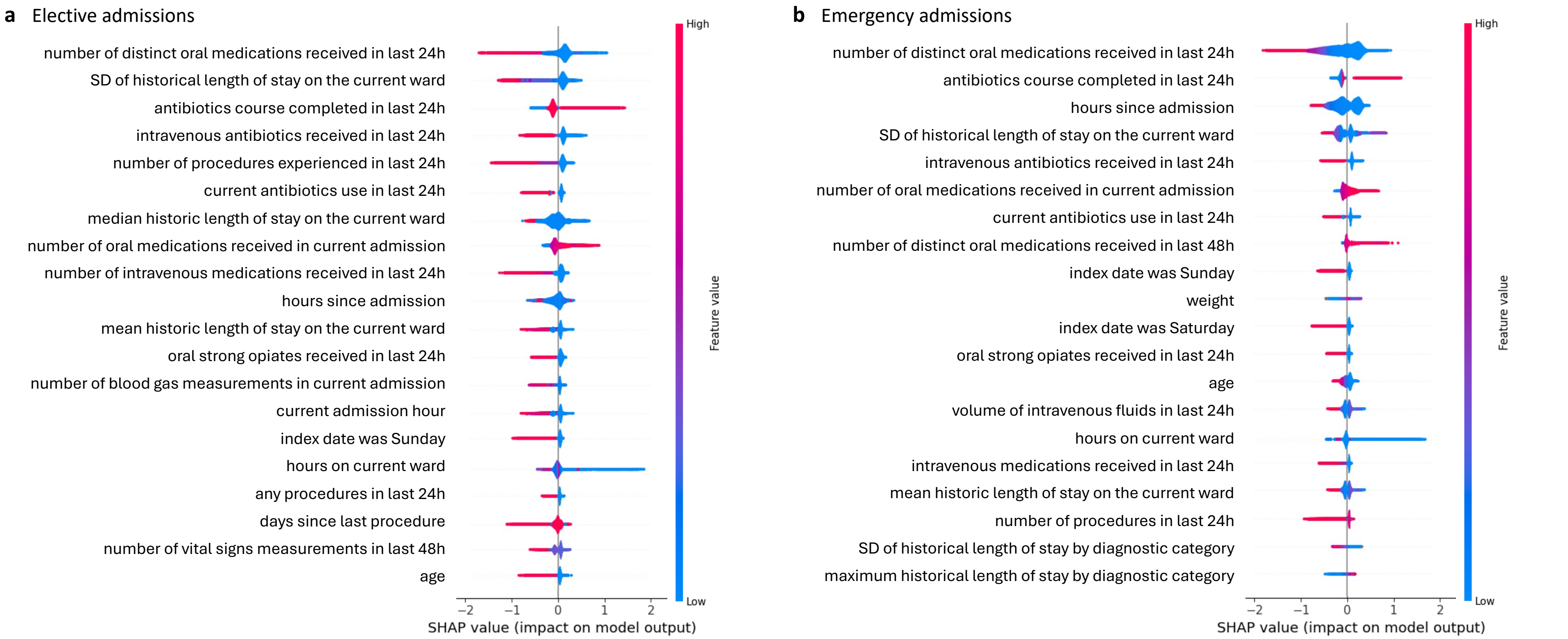

Supplementary Figure 12. Direction of association between the top 20 most predictive features and discharge using SHAP values for elective (a) and emergency (b) admissions. Negative SHAP value indicates less likely to be discharged, while positive SHAP value indicates more likely to be discharged.

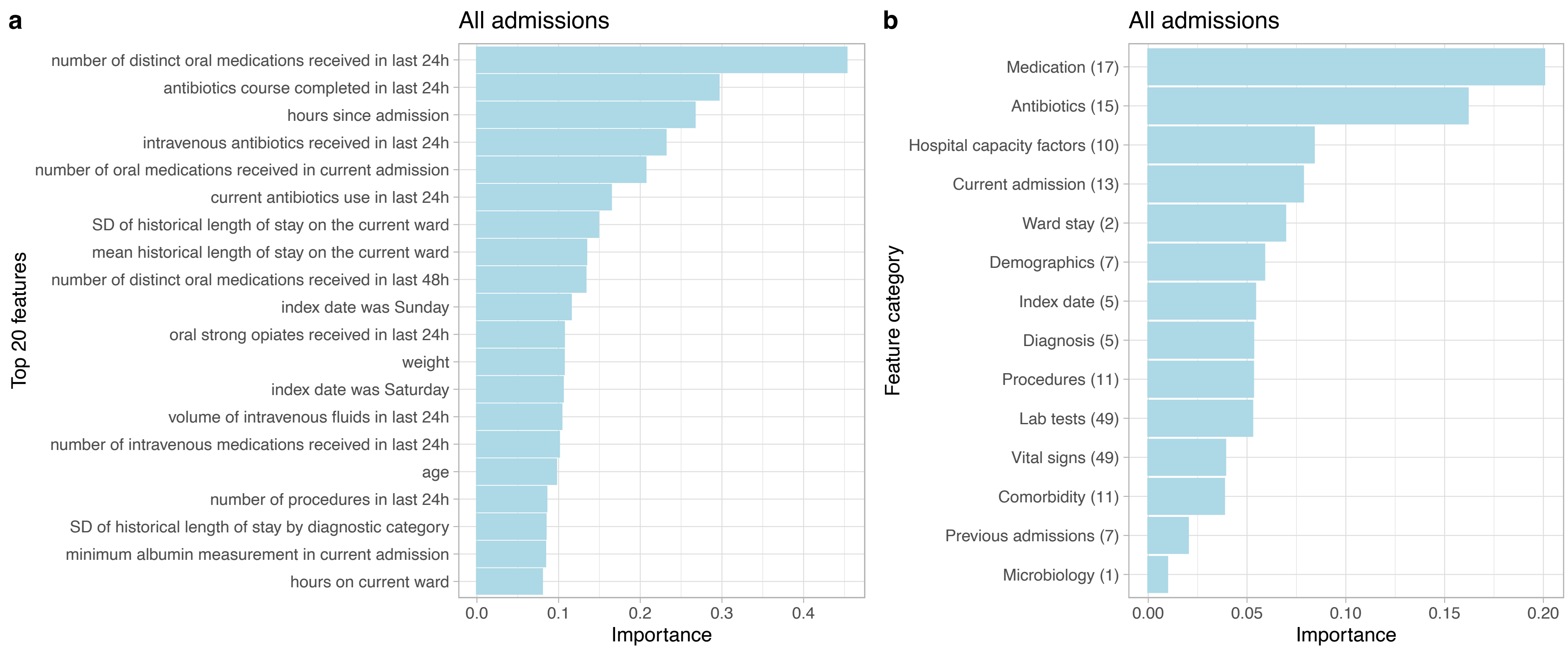

Supplementary Figure 13. Feature importance from the extreme gradient boosting model using SHAP values for all admissions, elective and emergency combined. The top 20 most predictive features are shown in the order of predictiveness in panel **a**. Feature importance grouped by feature category is shown in the order of predictiveness in panel **b.** The mean importance of the top 5 most important features within each category is plotted. Numbers shown in parenthesis are number of features within the top 200 most predictive features in each category. No discharge planning features were selected. The complete list of features is summarised in **Supplementary Table 1.** SHAP: SHapley Additive exPlanations. SD: standard deviation; LOS: length of stay; Current admission: admission time/source/specialty; SHMI: length of stay characteristics of SHMI diagnosis categories; Previous admissions: previous length of stay and readmission; Discharge: discharge planning.

Supplementary Tables

Reference	Publication/Year	Features
7	Safavi et al. 2019	Demographics, surgery information, clinician orders, clinical test results, bedside assessments, clinical recommendations, medication administration, catheter information, care team notes
8	Lazar et al. 2020	Age, sex, admission source, laboratory measurements, and vitals
9	Ahn et al. 2021	Index date-related features, diagnosis, operations, medications, procedures, laboratory tests, past medical history (last 3 years)
10	Zhang et al. 2021	Age, race, gender, insurance, user-EHR interactions (e.g., view/modify/export EHR entries), past medical history (Phecodes), discharge units, length of stay, discharge time, discharge day of week
11	Barnes et al. 2016	Gender, ethnicity, age, insurance, reason for visit, observation status, discharge location, patient census, day of week, elapsed length of stay
12	Levin et al. 2021	Demographic, administrative, temporal, medication, other interventions, diagnostics, monitoring, rehabilitation, consults, diet and more complex clinical markers (pain management, substance abuse, sepsis, cardiac arrest, acute kidney injury)
13	Bertsimas et al. 2021	Diagnosis, medications, laboratory results, body mass index, type of diet, level of activity and autonomy, socioeconomic factors, operations, laboratory results, and vitals
14	Ward et al. 2021	Age, gender, admit type, admission and hourly LAPS2 and COPS2 scores, diagnoses, hourly number of orders, medications, time since admission, do-not-resuscitate or comfort care

Supplementary Table 1. Features used in previous studies predicting discharge within a fixed time window. We searched Google Scholar and PubMed for studies up to 30 April 2024, using the search terms 'machine learning' AND ('hospital discharge prediction', OR 'patient flow'). AUROC: area under the receiver operating curve. EHR: electronic health records. LAPS2: Laboratory-based Acute Physiology Score, version 2. COPS2: Comorbidity Point Score, version 2. Population, outcome, model, and performance of each study are shown in **Table 1**.

Hyperparameter	Elective	Emergency	Overall	Optimisation options
**colsample_bytree**	0.5	0.8	0.4	0.2, 0.3, 0.4, 0.5, 0.6, 0.7, 0.8, 0.9, 1
**gamma**	0	0.3	0.4	0.1, 0.2, 0.3, 0.4, 0.5
**learning_rate**	0.05	0.05	0.2	0.001, 0.005, 0.01, 0.05, 0.1, 0.2, 0.5, 1
**max_depth**	7	7	6	3, 4, 5, 6, 7, 8, 9, 10, 11, 12
**min_child_weight**	2	3	2	1, 2, 3, 4, 5, 6
**n_estimators**	775	750	400	50, 75, 100, 125, …, 1200
**reg_alpha**	0.01	0.01	0.0001	0.0001, 0.001, 0.01, 0.1, 1, 10, 100
**reg_lambda**	0.01	10	100	0.0001, 0.001, 0.01, 0.1, 1, 10, 100
**subsample**	0.9	0.9	0.9	0.3, 0.4, 0.5, 0.6, 0.7, 0.8, 0.9, 1

Supplementary Table 2. Hyperparameters chosen by the extreme gradient boosting models.

Admission type	Data	Accuracy	Balanced accuracy	Sensitivity/Recall	Specificity	PPV/Precision	NPV	F1-score	AUC	AUPRC	MAE (%)
**Elective**	train	0.930	0.938	0.951	0.926	0.757	0.987	0.843	0.981	0.918	4.3
**Elective**	validation	0.835	0.790	0.718	0.863	0.558	0.927	0.628	0.889	0.676	16.6
**Elective**	test	0.823	0.767	0.673	0.861	0.555	0.911	0.609	0.871	0.658	8.9
**Emergency**	train	0.901	0.842	0.753	0.932	0.693	0.948	0.721	0.950	0.792	3.6
**Emergency**	validation	0.854	0.757	0.609	0.904	0.566	0.918	0.587	0.866	0.638	8.0
**Emergency**	test	0.844	0.756	0.616	0.896	0.571	0.912	0.593	0.860	0.644	4.9
**All**	train	0.869	0.797	0.686	0.907	0.612	0.931	0.647	0.911	0.704	3.5
**All**	validation	0.846	0.765	0.639	0.891	0.561	0.919	0.597	0.872	0.643	7.2
**All**	test	0.837	0.752	0.615	0.888	0.561	0.909	0.587	0.858	0.634	4.6

Supplementary Table 3. Model performance of the extreme gradient boosting (XGB) model predicting 24-hour discharge in the training, validation, and test dataset. PPV: positive predictive value; NPV: negative predictive value; AUC: area under the receiver operating curve; AUPRC: area under the precision-recall curve; MAE: normalised mean absolute error (mean difference in predicted and actual discharges per day divided by the mean number of discharges per day).
